# Correlation analysis of pharmacokinetic parameters of docetaxel AUC and adverse reactions in breast cancer patients

**DOI:** 10.3389/fphar.2025.1563506

**Published:** 2025-05-09

**Authors:** Yongzhe Tang, Yamin Liu, Shengying Qin, Cong Huai, Jin Zhang, Weijie Ding, Junwei Fan, Jie Wang, Xiaoqing Zhang

**Affiliations:** ^1^ Department of Breast Surgery, The International Peace Maternity and Child Health Hospital, School of Medicine, Shanghai Jiao Tong University, Shanghai, China; ^2^ Shanghai Key Laboratory of Embryo Original Diseases, Shanghai, China; ^3^ Department of Pharmacy, The International Peace Maternity and Child Health Hospital, School of Medicine, Shanghai Jiao Tong University, Shanghai, China; ^4^ Key Laboratory for the Genetics of Developmental and Neuropsychiatric Disorders (Ministry of Education), Bio-X Institutes, Shanghai Jiao Tong University, Shanghai, China; ^5^ Department of General Surgery, Shanghai General Hospital, School of Medicine, Shanghai Jiao Tong University, Shanghai, China

**Keywords:** Docetaxel, TDM (therapeutic drug monitoring), AUC (area under the curve), adverse reactions, breast cancer

## Abstract

**Background:**

Docetaxel is commonly used in breast cancer chemotherapy. The previous drug dose is generally calculated based on body surface area (BSA). However, the metabolism varies greatly among different patients. Docetaxel therapeutic drug monitoring (TDM) helps monitor adverse drug reactions and explore the appropriate range of area under the curve (AUC) to ensure chemotherapy effectiveness and reduce adverse reaction occurrence.

**Methods:**

We conducted a real-world retrospective study and included 180 breast cancer patients, who received a chemotherapy regimen containing docetaxel. The patients’ demographic and tumor data were reviewed. Adverse reaction data during chemotherapy treatment were collected through patient questionnaires and laboratory test results. Univariate logistic regression analysis was performed on 33 patient indexes, including basic information, blood toxicity, liver and kidney function, gastrointestinal reactions, and cardiotoxicity.

**Results:**

The adverse reactions of chemotherapy were matched with different docetaxel AUC results through univariate analysis. The patients between the groups were no statistically significant differences in terms of demographic and tumor data, including age, height, weight, BSA, and body mass index (*p* > 0.05). Univariate analysis revealed significant differences in albumin (ALB) levels (*p* = 0.037), creatinine (CREA) levels (*p* = 0.002), nausea occurrence (*p* = 0.008), vomiting occurrence (*p* = 0.013), rashes occurrence (*p* = 0.002), and chemotherapy-induced alopecia incidence (CIA) (*p* = 0.002). Based on the results of the univariate analysis, binary logistic regression analysis was further conducted to identify predictors contributing to the occurrence of chemotherapy adverse reactions. The results demonstrated that an AUC value greater than 2.5 mg h/L was significantly associated with increased risk of certain adverse reactions such as rashes, CIA, CREA, and ALB.

**Conclusion:**

The docetaxel TDM provides a reliable basis for monitoring chemotherapy adverse reactions, with high AUC significantly associated with certain adverse reactions. Future studies are expected to include more patients and conduct multi-center trials to obtain a suitable AUC range for Chinese patients, which will guide the determination of clinical chemotherapy doses and reduce the occurrence of adverse reactions.

## 1 Introduction

Adjuvant chemotherapy is a cornerstone in the treatment of breast cancer, aiming to reduce recurrence and increase overall survival in patients. Currently, standard adjuvant chemotherapy should include anthracyclines, taxanes, and cyclophosphamide (Braybrooke et al., 2023; Crown et al., 2004). Among taxanes, docetaxel is a highly active chemotherapeutic agent that is widely used not only in patients with early-stage breast cancer but also as an effective option for treating patients with metastatic breast cancer (Lyseng-Williamson and Fenton, 2005). Guided by the concept of chemotherapy downgrading, the combination of docetaxel and cyclophosphamide (TC) has been proven to be a preferred nonanthracyline-based regimen for reducing anthracycline cardiac toxicity in patients with node-negative and lower-risk node-positive breast cancer (Jones et al., 2009). For adjuvant therapy of human epidermal growth factor receptor 2 (HER2) positive breast cancer, combinations of docetaxel and trastuzumab (and pertuzumab) demonstrate synergistic cytotoxic activity while also being favorable from both safety and efficacy perspectives (Burris, 2001).

Docetaxel, a narrow therapeutic index (NTI) drug, is primarily metabolized through the hepatobiliary pathway and excreted in feces. As a NTI drug, docetaxel exhibits a narrow effective internal concentration range that closely approaches the toxic concentration range. This characteristic can result in significant treatment failure or serious adverse reactions due to minimal differences in dosage or blood concentration levels. Currently, the dose of docetaxel in clinical practice is generally calculated according to the patient’s body surface area (BSA), but taking this dosage alone may result in a concentration difference of up to 7-fold between individuals (Beumer et al., 2012). On the other hand, with the expiration of its patent, both the original drug and generic versions from multiple manufacturers are used in clinical practice. Variations among different brands, formulations, and individual patients may lead to substantial differences in internal concentrations and increase the risk of adverse effects. Numerous studies have demonstrated that even minor changes in excipients, solvents (such as polysorbate 80 and ethanol), or unbound fractions of docetaxel are associated with severe hematological toxicities including febrile neutropenia and cutaneous toxicities (Chen and Huang, 2021).

Variations in docetaxel metabolism among patients may lead to high blood concentrations and increased risk of adverse reactions. Balancing the efficacy of chemotherapy with the reduction of adverse reactions poses a challenge. Therapeutic drug monitoring (TDM) is a new approach that applies modern analytical techniques (Pouliquen et al., 2011). The area under the curve (AUC) of docetaxel can be used to assess whether adverse effects are attributable to drug overdose, minimize adverse effects while maintaining treatment efficacy, and provide a basis for necessary dose adjustments. Therefore, this study aims to assist with docetaxel dose adjustment and adverse effect management through docetaxel TDM.

## 2 Materials and methods

### 2.1 Patients

A total of 180 breast cancer patients treated at the Department of Breast Surgery, International Peace Maternity and Child Health Hospital (IPMCH) between 26 August 2022, and 28 June 2024, were included in this study. Other eligibility criteria were as follows: (1) patients underwent radical surgery for breast cancer and received adjuvant chemotherapy with a docetaxel-based regimen, (2) Eastern Cooperative Oncology Group (ECOG) performance status of 0–1, (3) an expected survival time of ≥3 months, (4) normal function of major organs, (5) voluntary participation with signed informed consent, (6) good treatment compliance, (7) willingness to cooperate during follow-up. Exclusion criteria were as follows: (1) patients were excluded if they exhibited poor treatment adherence, (2) significant comorbidities involving major organs, (3) known allergies to taxane drugs or their excipients, (4) severe or uncontrolled infections, (5) a history of substance abuse or mental disorders, (6) pregnancy or breastfeeding, (7) patients with incomplete medical records were excluded. The Ethics Committee of IPMCH approved this study (No. GKLW-2022-34), and written informed consent was obtained from all enrolled patients.

We conducted a comprehensive analysis of the patients’ demographic characteristics, encompassing age, height, weight, BSA, and body mass index (BMI). Additionally, we thoroughly examined the patients’ tumor-related data, including pathological TNM staging and molecular classification. The molecular classification of patients was based on the differential expression levels of estrogen receptor (ER), progesterone receptor (PR), HER2. The establishment of chemotherapy regimens for patients followed the recommendations outlined in NCCN guidelines and CACA-CBCS guidelines (Gradishar et al., 2024; The Society of Breast Cancer China Anti-Cancer Association, Breast Oncology Group of the Oncology Branch of the Chinese Medical Association 2023). The recommended dosing regimen for docetaxel is typically administered every 3 weeks for four to six cycles, at a dose of 75 mg/m^2^ (Dimitrakopoulos et al., 2024). Pegylated human granulocyte colony-stimulating factor (peg-G-CSF) was administered to high-risk patients as a prophylactic measure against severe neutropenia. Dexamethasone, parosetron, magnesium isoglycyrrhizate, and/or reductive glutathione were utilized for the prevention of chemotherapy-induced allergic reactions, vomiting, and hepatotoxicities.

### 2.2 Docetaxel TDM

All subjects were treated with docetaxel by intravenous infusion. Administered at a dose of 75 mg/m^2^ according to docetaxel drug dosing guidelines. Docetaxel was diluted with 250 mL of 0.9% sodium chloride injection or 5% glucose injection solvent and the infusion was completed within 1 h. Peripheral blood samples were collected in heparinized tubes 0-10 minutes before the end of the infusion and 60 minutes after infusion completion, collected in an anticoagulant tube containing heparin at 4 °C, followed by centrifugation (3,000 rpm, 10 min at 4°C), and then the supernatant was taken and placed at −80 °C for further analysis. Subsequent analysis is thawed on ice to prevent any possible changes in the sample, and drug extraction is carried out by protein precipitation, centrifugation and other steps. The processed samples are finally analyzed by high-performance liquid chromatography mass spectrometry. The AUC value was calculated by Mycare™ drug exposure calculation software according to docetaxel dose, intravenous infusion start and end time, number of blood collections, and docetaxel blood concentration.

In this study, the systemic clearance (CL) of docetaxel was calculated by integrating pharmacokinetic model parameters with Bayesian analysis. The Bayesian analysis, performed using the Mycare™ drug exposure calculation software, incorporated individualized patient data (e.g., drug concentrations, physiological parameters) to refine CL estimation accuracy. Subsequently, the total drug exposure (AUC_0-inf_) was estimated using the classical pharmacokinetic formula AUC = D/CL, which reflects the total systemic exposure from drug administration to complete elimination by relating the administered dose (D) to the CL.

### 2.3 Adverse reactions

During chemotherapy treatment, adverse reaction data were collected through the administration of adverse reaction questionnaires and laboratory test results, encompassing hematologic toxicity, hepatotoxicities, renal toxicity, gastrointestinal reactions, canker sores, finger numbness, skin rashes, electrocardiogram (ECG) abnormalities and chemotherapy-induced alopecia (CIA). The grading of patients’ adverse reactions was conducted in accordance with CTCAE 5.0 standards.

### 2.4 Statistical analysis

Categorical variables are expressed as percentages, and continuous variables are expressed as mean (± standard deviation, SD) or interquartile ranges. Univariate logistic regression analysis was used to analyze 33 indexes of patients, including patients’ demographic, tumor data and adverse reactions. The index of *p* < 0.05 in univariate analysis was selected for further multivariate logistic regression analysis. The data were analyzed using SPSS software (version 26.0, IBM, Armonk, NY, United States of America), and GraphPad Prism software (version 9.0, La Jolla, CA, United States of America) was used to analyze and plot AUC values and indicators. *P < 0*.05 indicated that the difference was statistically significant (*, *P < 0*.05; **, *P* < 0.01).

## 3 Results

### 3.1 Patient characteristics

The 180 patients included in this study all received chemotherapy regimens containing docetaxel, and the patient characteristics are presented in Table 1. The median age of the patients was 54.5 years (range: 46–62 years). The median BSA was calculated to be 1.64 m^2^, and the BMI averaged at 23.44 kg/m^2^. A majority of the patients were diagnosed with early-stage breast cancer, with stage I and stage II patients accounting for 91.67% of the total. A classic TC regimen (docetaxel and cyclophosphamide) was administered to 50% of the patients, while anthracycline-containing regimens were used in 16% of cases. Additionally, carboplatin-containing regimens were used for treating HER2-positive and triple negative breast cancer (TNBC) patients at a rate of approximately 25.6%, whereas HER2-targeted therapy with trastuzumab (or trastuzumab-pertuzumab in axillary lymph nodes positive patients) was given to 30% of HER2-positive cases. Notably, only one patient with metastases received a maintenance regimen consisting of docetaxel combined with Tyrosine Kinase Inhibitor (TKI) anti-HER2-targeted therapy.

**TABLE 1:** Demographic and clinical data of all patients.

Parameter	Median (interquartile range)/Number (%)
*Characteristics*	
Age (year)	54.5 [46, 62]
Height (cm)	160 [156.5, 164]
Weight (kg)	60 [55, 65]
BSA (m^2^)	1.64 [1.57, 1.71]
BMI (kg/m^2^)	23.44 [21.32, 25.37]
*T*	
T1	118 (65.56)
T2	55 (30.56)
T3	6 (3.33)
T4	1 (0.56)
*N*	
N0	129 (71.67)
N1	41 (22.78)
N2	5 (2.78)
N3	5 (2.78)
*M*	
M0	179 (99.44)
M1	1 (156)
*P*athologic *stage*	
I	95 (52.78)
II	70 (38.89)
III	14 (7.78)
IV	1 (0.56)
*Molecular Classification*	
Luminal A	44 (24.44)
Luminal B	91 (50.56)
HER-2 (+)	31 (17.22)
TNBC	14 (7.78)
*Chemotherapy regimens*	
TC	90 (50.00)
TCb	1 (0.56)
TCbH	16 (8.89)
TCbHP	29 (16.11)
TCH	12 (6.67)
TEC	8 (4.44)
EC-T	15 (8.33)
EC-TH	1 (0.56)
EC-THP	6 (3.33)
THP	1 (0.56)
TH-Pyrotinib	1 (0.56)

Abbreviations: BSA, body surface area; BMI, body mass index; *T*, tumor; *N*, regional lymph node; *M*, metastasis; HER-2, human epidermal growth factor receptor 2; TNBC, triple-negative breast cancer; E, epirubicin; C, cyclophosphamide; T, docetaxel; b, Carboplatin; H, herceptin; P, perjeta.

### 3.2 AUC and adverse toxicity analysis

All patients received docetaxel TDM, and classified into three groups based on the range of AUC reference values (AUC <1.5 mg h/L, 1.5 mg h/L ≤ AUC ≤2.5 mg h/L, AUC >2.5 mg h/L). Demographic characteristics of the patients including age, height, weight, BSA, and BMI were recorded along with tumor-related data such as pathological TNM staging and molecular classification by groups in Table 2. The results indicated that the fundamental characteristics of the patients were relatively equilibrated across all groups.

**TABLE 2:** Analysis of AUC data in patients with docetaxel chemotherapy.

Group (mg·h/L)	AUC <1.5	1.5 ≤ AUC ≤2.5	AUC >2.5
Mean (range)/Number (%)	53 (29.44)	52 (34.44)	75 (36.11)
***Age(year)***	53.38 (29–73)	52.98 (37–72)	54.77 (37–74)
***Height (cm)***	160.39 (148–179)	160.17 (153–173)	159.87 (148–171)
***Weight (kg)***	60.39 (48–75)	60.28 (45–90)	59.71 (41–81)
***BSA (m*** ^***2***^ ***)***	1.65 (0.84–2.32)	1.66 (1.44–2.44)	1.64 (1.38–1.95)
***BMI (kg/m*** ^***2***^ ***)***	23.54 (16.23–28.40)	23.66 (19.14–35.16)	23.34 (16.85–30.48)
*T*			
T1	37 (69.81)	32 (61.54)	49 (65.33)
T2	15 (28.30)	18 (34.62)	22 (29.33)
T3	1 (1.89)	2 (3.85)	3 (4.00)
T4	-	-	1 (1.33)
*N*			
N0	38 (71.70)	36 (69.23)	55 (73.33)
N1	13 (24.53)	11 (21.15)	17 (22.67)
N2	2 (3.77)	2 (3.85)	1 (1.33)
N3	-	3 (5.77)	2 (2.67)
***M***			
M0	53 (100)	52 (100)	74 (98.67)
M1	-	-	1 (1.33)
*Pathologic stage/N (%)*			
I	30 (56.60)	27 (51.92)	39 (52.00)
II	20 (37.74)	19 (36.54)	30 (40.00)
III	3 (5.66)	6 (11.54)	5 (6.67)
IV	-	-	1 (1.33)
*Molecular Classification*			
Luminal A	13 (24.53)	11 (21.15)	20 (26.67)
Luminal B	26 (49.06)	28 (53.85)	38 (50.67)
HER-2 (+)	8 (15.09)	10 (19.23)	12 (16.00)
TNBC	6 (11.32)	3 (5.77)	5 (6.67)
*Chemotherapy regimens*			
TC	23 (43.40)	25 (48.08)	42 (56.00)
TCb	-	1 (1.92)	-
TCbH	8 (15.09)	4 (7.69)	4 (5.33)
TCbHP	7 (13.21)	7 (13.46)	15 (20.00)
TCH	3 (5.66)	4 (7.69)	5 (6.67)
TEC	1 (1.89)	6 (11.54)	1 (1.33)
EC-T	8 (15.09)	3 (5.77)	4 (5.33)
EC-TH	-	1 (1.92)	-
EC-THP	2 (3.77)	1 (1.92)	3 (4.00)
THP	1 (1.89)	-	-
TH-Pyrotinib	-	-	1 (1.33)

Abbreviations: AUC, area under the concentration–time curve; BSA, body surface area; BMI, body mass index; *T*, tumor; *N*, regional lymph node; *M*, metastasis; HER-2, human epidermal growth factor receptor 2; TNBC, triple-negative breast cancer; E, epirubicin; C, cyclophosphamide; T, docetaxel; b, Carboplatin; H, herceptin; P, perjeta.

We collected detailed adverse reaction data from patients using the patients’ adverse reaction questionnaire and laboratory tests. The grading of patients’ adverse reactions was based on the CTCAE 5.0 standards, wherein Grade 0 denoted absence of adverse events and Grade 4 represented the most severe adverse reactions in Table 3. Despite some high-risk patients receiving preventive supportive care with peg-G-CSF, the grade 4 adverse reactions primarily consisted of leukopenia (3 cases) and chemotherapy-induced neutropenia (CIN) (54 cases), while other grade 4 adverse reactions mainly included gastrointestinal symptoms (1 case of vomiting, 2 cases of diarrhea, and 5 cases of constipation). Grade 2/3 adverse reactions encompassed thrombocytopenia, hepatotoxicities, gastrointestinal symptoms (nausea, vomiting, diarrhea, constipation), oral ulcers, finger numbness, skin rashes, and CIA.

**TABLE 3:** Adverse reactions of chemotherapy at each stage of the patients.

Adverse events	Grade 0/Normal	Grade I/Abnormal	Grade II	Grade III	Grade IV
*Hematological toxicities*					
RBC (×10^12^/L)	74 (41.11)	106 (58.89)	-	-	-
HGB (g/L)	64 (35.56)	116 (64.44)	-	-	-
Leukocyte (×10^9^/L)	67 (37.22)	24 (13.33)	39 (21.67)	47 (26.11)	3 (1.67)
Neutrophil (×10^9^/L)	58 (32.22)	23 (12.78)	18 (10)	27 (15)	54 (30)
PLT (×10^9^/L)	142 (78.89)	26 (14.44)	8 (4.44)	4 (2.22)	-
*Hepatotoxicities*					
ALT (U/L)	85 (47.22)	77 (42.78)	17 (9.44)	1 (0.56)	-
AST (U/L)	110 (61.11)	63 (35)	1 (0.56)	6 (3.33)	
GGT (U/L)	110 (61.11)	70 (38.89)	-	-	-
ALP (U/L)	158 (87.78)	22 (12.22)	-	-	-
ALB (g/L)	157 (87.22)	23 (12.78)	-	-	-
TBIL (μmol/L)	156 (86.67)	24 (13.33)	-	-	-
DBIL (μmol/L)	157 (87.22)	23 (12.78)	-	-	-
*Renal toxicities*					
UREA (μmol/L)	138 (76.67)	42 (23.33)	-	-	-
CREA (μmol/L)	129 (71.67)	51 (28.33)	-	-	-
UA (μmol/L)	82 (45.56)	98 (54.44)	-	-	-
*Gastrointestinal reactions*					
Nausea	65 (36.31)	79 (44.13)	16 (8.94)	19 (10.61)	-
Vomiting	127 (70.95)	41 (21.91)	8 (4.47)	2 (1.12)	1 (0.56)
Diarrhea	125 (69.83)	36 (20.11)	13 (7.26)	3 (1.68)	2 (1.12)
Constipation	120 (67.04)	33 (18.44)	10 (5.59)	11 (6.15)	5 (2.79)
*Others*					
Canker sores	138 (77.09)	30 (16.76)	7 (3.91)	4 (2.23)	-
Finger Numbness	133 (74.30)	43 (24.02)	3 (1.68)	-	-
Rashes	140 (78.21)	20 (11.17)	12 (6.70)	7 (3.91)	-
ECG	88 (48.89)	92 (51.11)	-	-	-
CIA	22 (12.22)	35 (19.44)	123 (68.33)	-	-

Abbreviations: RBC, red blood cell; HGB, hemoglobin; PLT, platelet; ALT, alanine aminotransferase; AST, aspartate aminotransferase; GGT, γ-glutamyl transpeptidase; ALP, alkaline phosphatase; ALB, albumin; TBIL, total bilirubin; DBIL, direct bilirubin; CREA, creatinine; UA, uric acid; ECG, electrocardiogram; CIA, chemotherapy-induced alopecia.

**FIGURE 1:**
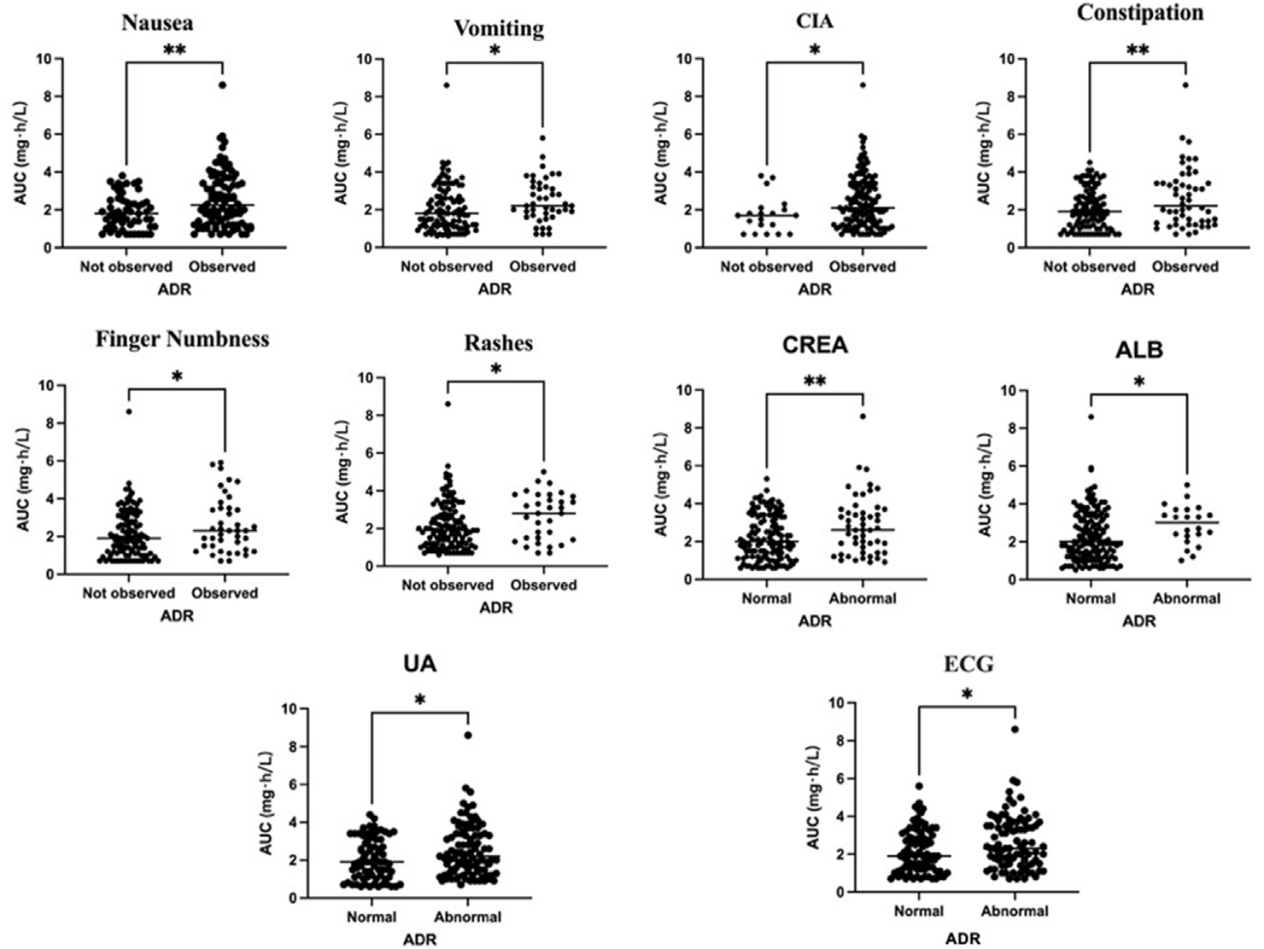
Relationship between docetaxel AUC exposure and adverse effects.

The docetaxel AUC and adverse reactions were analyzed using univariate analysis, and the significant factors identified in the results were presented (Figure 1). It was observed that patients experiencing vomiting, CIA, finger numbness, rashes, abnormal albumin (ALB), abnormal uric acid (UA), or abnormal ECG exhibited a higher AUC compared to those without adverse reactions, with statistical significance (*P <* 0.05). Furthermore, patients with nausea, constipation, and abnormal creatinine (CREA) also demonstrated a significantly higher AUC compared to those who did not exhibit these symptoms (*P <* 0.01).

In order to further investigate the association between adverse reactions and docetaxel AUC, as well as explore a more appropriate range for AUC, the included patients were divided into two groups based on an upper limit of 2.5 mg h/L for AUC. Among them, there were 105 patients in the group with AUC ≤2.5 mg h/L and 75 patients in the group with AUC >2.5 mg h/L. The univariate analysis was divided into analyzing the relationship between AUC and continuous variables and the relationship between categorical variables (Tables 4 and 5). The results revealed no significant differences in age, height, weight, BSA, BMI or other basic characteristics between the two groups (*p* > 0.05). However, univariate regression analysis of adverse reactions demonstrated statistically significant differences between the two groups in terms of ALB (*p* = 0.037), CREA (*p* = 0.002), gastrointestinal reactions (nausea [*p* = 0.008] and vomiting [*p* = 0.013]), rashes (*p* = 0.002), as well as CIA (*p* = 0.002).

**TABLE 4:** Demographic and linear regression analysis of continuous variables.

Variable	AUC grade (mg·h/L)				Univariate regression analysis	
	AUC ≤2.5 (*n* = 105)		AUC >2.5 (*n* = 75)		OR (95%CI)	*P-*value
	Mean (SD)	Median (range)	Mean (SD)	Median (range)		
**Age (year)**	53.29 (10.58)	52 (29–73)	54.77 (9.32)	57 (37–74)	1.015 (0.985–1.046)	0.328
**Height (cm)**	160.33 (5.33)	160 (148–179)	159.87 (5.46)	160 (148–171)	0.984 (0.931–1.040)	0.569
**Weight (kg)**	60.33 (7.81)	60.00 (45.00–90.00)	59.71 (7.82)	60.00 (41–81)	0.990 (0.952–1.028)	0.593
**BSA (m** ^**2**^ **)**	1.65 (0.17)	1.64 (0.84–2.44)	1.64 (0.12)	1.64 (1.38–1.95)	0.588 (0.077–4.497)	0.609
**BMI (kg/m** ^**2**^ **)**	23.57 (2.84)	23.63 (16.23–35.16)	23.34 (2.68)	23.14 (16.85–30.48)	0.970 (0.871–1.081)	0.586
**RBC (×10** ^**12**^ **/L)**	3.76 (0.64)	3.69 (2.17–5.05)	3.93 (0.74)	3.73 (2.29–6.45)	1.442 (0.924–2.251)	0.107
**HGB (g/L)**	111.45 (17.96)	109.00 (65.00–150.00)	114.55 (14.94)	112.00 (80.00–151.00)	1.011 (0.993–1.030)	0.223
**Leukocyte (×10** ^**9**^ **/L)**	3.78 (2.51)	2.70 (0.8–9.5)	3.67 (2.36)	2.80 (0.80–9.30)	0.981 (0.868–1.108)	0.754
**Neutrophil (×10** ^**9**^ **/L)**	1.83 (1.88)	1.05 (0.03–6.57)	1.79 (1.87)	0.97 (0.01–6.50)	0.990 (0.844–1.161)	0.899
**PLT (×10** ^**9**^ **/L)**	199.37 (89.71)	194.00 (33.00–542.00)	213.21 (79.64)	207.00 (54.00–432.00)	1.002 (0.998–1.005)	0.286
**ALT (U/L)**	47.90 (39.74)	40.00 (8.00–286.00)	39.45 (42.74)	29.00 (7.00–338.00)	0.994 (0.986–1.003)	0.186
**AST (U/L)**	37.63 (28.02)	27.50 (13.00–221.00)	33.70 (31.20)	23.00 (10.00–230.00)	0.995 (0.984–1.006)	0.372
**GGT (U/L)**	65.26 (71.97)	38.00 (10.00–393.00)	54.67 (55.12)	40.00 (10.00–310.00)	0.997 (0.993–1.002)	0.289
**ALP (U/L)**	68.88 (27.84)	64.00 (31.00–207.00)	73.91 (23.12)	69.00 (40.00–170.00)	1.007 (0.996–1.019)	0.207
**ALB (g/L)**	42.95 (5.05)	42.00 (28.80–67.00)	44.81 (6.54)	44.00 (33.70–70.00)	1.059 (1.003–1.118)	0.037*
**TBIL (μmol/L)**	8.69 (5.95)	6.80 (2.00–36.30)	7.95 (7.25)	6.30 (1.50–56.80)	0.981 (0.935–1.031)	0.453
**DBIL (μmol/L)**	2.55 (2.35)	1.90 (0.60–18.40)	2.85 (2.35)	2.10 (0.40–15.20)	1.057 (0.931–1.201)	0.391
**UREA (μmol/L)**	5.51 (2.00)	5.30 (2.10–10.40)	5.38 (2.10)	5.20 (1.60–12.90)	0.969 (0.837–1.122)	0.674
**CREA (μmol/L)**	61.26 (13.44)	60.00 (36.00–105.65)	70.30 (22.86)	67.00 (40.41–170.93)	1.030 (1.011–1.049)	0.002**
**UA (μmol/L)**	316.30 (92.85)	326.40 (129.00–498.70)	339.70 (115.33)	344.50 (139.70–596.80)	1.002 (0.999–1.005)	0.135

Abbreviations: BSA, body surface area; BMI, body mass index; RBC, red blood cell; HGB, hemoglobin; PLT, platelet; ALT, alanine aminotransferase; AST, aspartate aminotransferase; GGT, γ-glutamyl transpeptidase; ALP, alkaline phosphatase; ALB, albumin; TBIL, total bilirubin; DBIL, direct bilirubin; CREA, creatinine; UA, uric acid.

Note: Used to analyze the relationship between AUC, and continuous baseline characteristics or laboratory data. This part of the analysis is applicable to continuous variables such as age, height, and weight. *P < 0.05, **P < 0.01.

**TABLE 5:** Demographics and logistic regression analyses of categorical variables.

Variable	AUC grade (mg·h/L)		Univariate regression analysis	
	AUC ≤2.5 (n = 105)	AUC >2.5 (n = 75)	OR (95%CI)	*P-*value
	Number of cases (percentage)	Number of cases (percentage)		
Nausea				
Normal	44 (41.90%)	17 (22.67%)	2.461 (1.265–4.786)	0.008**
Abnormal	61 (58.10%)	58 (77.33%)		
Vomiting				
Normal	80 (76.19%)	44 (58.67%)	2.255 (1.186–4.287)	0.013*
Abnormal	25 (23.81%)	31 (41.33%)		
Diarrhea				
Normal	74 (70.48%)	53 (70.67%)	0.991 (0.517–1.899)	0.978
Abnormal	31 (29.52%)	22 (29.33%)		
Constipation				
Normal	74 (70.48%)	44 (58.67%)	1.682 (0.903–3.133)	0.101
Abnormal	31 (29.52%)	31 (41.33%)		
Canker				
Normal	79 (75.24%)	60 (80.00%)	0.760 (0.370–1.559)	0.453
Abnormal	26 (24.76%)	15 (20.00%)		
Finger Numbness				
Normal	79 (75.24%)	55 (73.33%)	1.105 (0.561–2.174)	0.773
Abnormal	26 (24.76%)	20 (26.67%)		
Rashes				
Normal	90 (85.71%)	49 (65.33%)	3.184 (1.543–6.570)	0.002**
Abnormal	15 (14.29%)	26 (34.67%)		
ECG				
Normal	51 (48.57%)	37 (49.33%)	0.970 (0.536–1.755)	0.920
Abnormal	54 (51.43%)	38 (50.67%)		
CIA				
Normal	43 (40.95%)	14 (18.67%)	3.022 (1.502–6.080)	0.002**
Abnormal	62 (59.05%)	61 (81.33%)		
*T*				
T1	69 (65.71%)	49 (65.33%)	1.131 (0.681–1.879)	0.634
T2	33 (31.42%)	22 (29.33%)		
T3	3 (2.86%)	3 (4.00%)		
T4	-	1 (1.33%)		
*N*				
N0	74 (70.48%)	55 (73.33%)	0.880 (0.561–1.379)	0.576
N1	24 (22.86%)	17 (22.67%)		
N2	4 (3.81%)	1 (1.33%)		
N3	3 (2.86%)	2 (2.67%)		
*M*				
M0	105 (100%)	74 (98.67%)	-	-
M1	-	1 (1.33%)		
*P stage*				
I	56 (53.33%)	39 (52.00%)	1.049 (0.670–1.643)	0.834
II	40 (38.10%)	30 (40.00%)		
III	9 (8.57%)	5 (6.67%)		
IV		1 (1.33%)		
*Molecular Classification*				
Luminal A	25 (23.81%)	20 (26.67%)	1	0.926
Luminal B	52 (49.52%)	38 (50.67%)	0.913 (0.444–1.880)	0.806
HER-2 (+)	19 (18.10%)	12 (16.00%)	0.789 (0.311–2.004)	0.619
TNBC	9 (8.57%)	5 (6.67%)	0.694 (0.201–2.403)	0.565
*Chemotherapy regimens*				
exclude carboplatin	78 (74.29%)	56 (74.67%)	0.980 (0.497–1.935)	0.954
include carboplatin	27 (25.71%)	19 (25.33%)		

Abbreviations: ECG, electrocardiogram; CIA, chemotherapy-induced alopecia; *T*, tumor; *N*, regional lymph node; *M*, metastasis; HER-2, human epidermal growth factor receptor 2; TNBC, triple-negative breast cancer.

Note: Used to analyze the relationship between AUC, and categorical variables.

**FIGURE 2:**
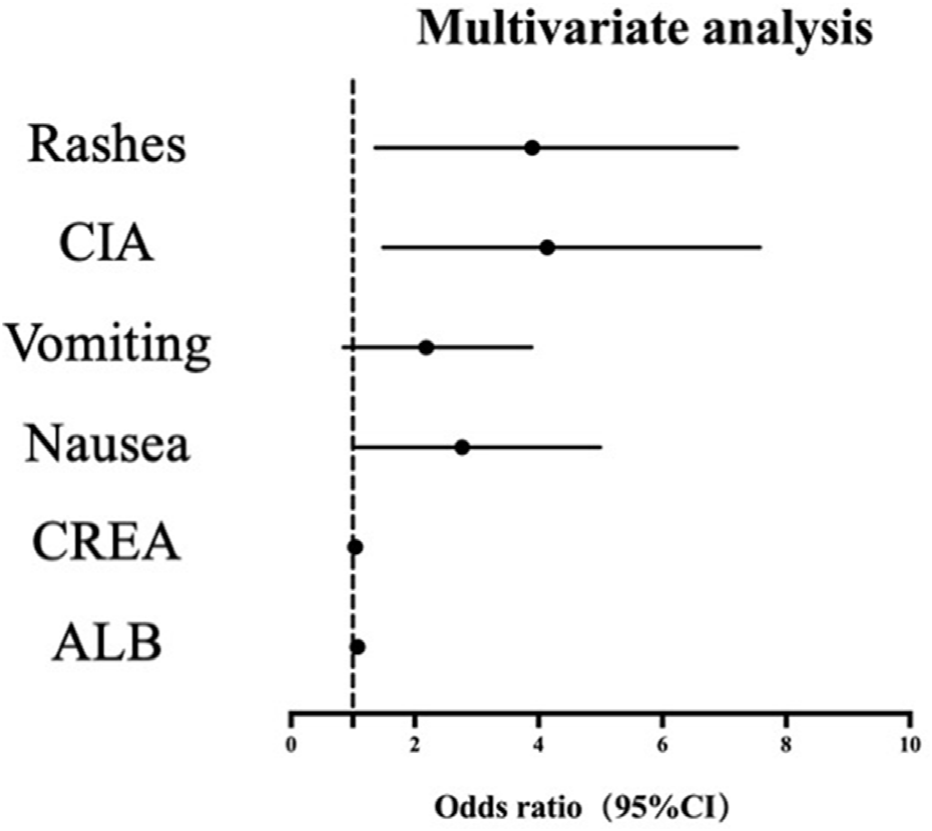
Nomogram of multivariate logistic regression analysis.

In the multivariate logistic regression analysis, we used the Benjamini–Hochberg method to correct the P values of each variable for multiple comparisons to control the false discovery rate (FDR). After correction, except for the P value of the variable “nausea (2.268 (1.029–5.000), *p* = 0.050) “which did not reach the significance level (P > 0.05), the adjusted P values of “rashes (OR 3.131 (1.363–7.196), *p* = 0.014)”, “CIA (OR 3.359 (1.489–7.575), *p* = 0.009)”, “CREA (OR 1.038 (1.016–1.061), *p* = 0.006)” and “ALB (OR 1.076 (1.010–1.146), *p* = 0.035)” were all less than 0.05, indicating that the association between these variables and AUC was statistically significant (Table 6). Figure 2 shows the corresponding binary logistic regression analysis nomogram.

**TABLE 6:** Multivariate logistic regression analysis (FDR corrected).

Variable	OR (95% CI)	*P* value	q value (FDR corrected)	Baseline Risk (%)	Adjusted Risk (%)	Risk difference (95% CI)
Rashes	3.131 (1.363–7.196)	0.007	0.014	14.29	34.30	+20.0 (8.1–31.2)
CIA	3.359 (1.489–7.575)	0.003	0.009	59.05	82.80	+23.8 (12.5–34.9)
Vomiting	1.820 (0.852–3.887)	0.122	0.122	23.81	36.20	+12.4 (−4.3–28.1)
Nausea	2.268 (1.029–5.000)	0.042	0.050	58.10	75.80	+17.7 (5.2–29.5)
CREA	1.038 (1.016–1.061)	0.001	0.006	-	-	Risk +3.8% per unit increase
ALB	1.076 (1.010–1.146)	0.023	0.035	-	-	Risk +7.6% per unit increase

^a^ Note:Risk difference confidence intervals were calculated using the Bootstrap method (1,000 resamples).

^b^ For continuous variables (CREA, ALB), the risk difference is approximated based on the OR, value (OR-1) and should be further validated with baseline risk data.

^c^ Baseline risk: Derived from the event incidence rate in the unexposed subgroup of this study cohort.

## 4 Discussion

TDM is a clinical practice that involves the measurement of specific drugs at designated intervals, aiming to optimize individual dosage regimens (Kang and Lee, 2009). In this study, docetaxel TDM utilized an advanced analytical technology, high-performance liquid chromatography-tandem mass spectrometry, to quantify the concentrations of drugs in patients’ biological fluids, monitor personalized variations in medication dosage and mitigate drug-induced adverse reactions. This study aims to investigate the correlation between the AUC values of docetaxel TDM and patient demographic and clinical data encompassing a total of nine indexes, as well as various adverse reactions associated with docetaxel chemotherapy encompassing a total of 24 indexes.

Initially, this study explored the correlation between the AUC values and nine indexes of patient demographic and clinical data. Previous studies have indicated that individual factors such as age, BMI, and BSA may be associated with adverse reactions to docetaxel. A small-scale Japanese study found significantly higher clearance rates of docetaxel among patients over 58 years old compared to younger individuals (Onoue et al., 2016). Another prospective study suggested no significant difference in docetaxel drug metabolism between elderly people over 65 years old and younger individuals, but it may lead to increased neutropenia (ten Tije et al., 2005). Literature also suggests that obese patients receiving docetaxel may not require dosage alterations, while a dosage reduction might be suitable for cyclophosphamide (Hall et al., 2013). Nevertheless, our results did not reveal any significant differences in patient demographic and clinical data among groups with different AUC values. This observation may be attributed to the majority of early-stage breast cancer patients enrolled in this study, as well as potential variations in population composition and tumor types reported in the existing literature. However, based on the literature findings, despite the continued use of BSA as the basis for calculating the dose of docetaxel in most patients today, elder patients and those with excessively high/low BMI still warrant attention. We eagerly anticipate new discoveries from larger sample studies in future research.

Another significant aspect of this study was to explore the correlation between the AUC and the evaluation of adverse reactions associated with docetaxel chemotherapy, encompassing a total of 24 items, such as hematologic toxicity, hepatotoxicities, renal toxicity, gastrointestinal reactions, canker sores, finger numbness, skin rashes, ECG abnormalities and CIA (Muth et al., 2021). Primary prevention strategies, such as the administration of pegfilgrastim (peg-G-CSF), are selected by therapists or patients based on specific chemotherapy regimens or significant risk factors for severe CIN (Li et al., 2017), and some other adjuvant drugs are commonly utilized to prevent vomiting or hepatic compromise in all chemo-supportive protocols. These measures may result in a reduction in the incidence of adverse drug reactions as well as a decrease in the severity of adverse effects.

The most common toxicities associated with docetaxel is hematologic toxicities, with CIN constituting a significant proportion. Febrile neutropenia (FN) defined as an oral temperature >38.5°C or two consecutive readings >38.0°C for 2 h with a severe neutropenia (ANC <500 cells/mm^3^), might be the most critical condition can increase risk of life-threatening infections (Fontanella et al., 2014). Severe CIN often leads to treatment delays and dose reductions in chemotherapy regimens. In this study, CIN was the most frequent adverse reaction, up to 67.78%. The incidence rates of Grade 3 (15%, 27cases) and Grade 4 (30%, 54 cases) CIN were found to be consistent with meta-analysis results involving breast cancer patients receiving TC chemotherapy. (Do et al., 2015). It is likely that the administration of peg-G-CSF in certain high-risk patients contributed to the lack of statistically significant differences between the AUC >2.5 mg h/L group and AUC ≤2.5 mg h/L group. Other hematologic toxicities were relatively mild, with 64.44% of patients experiencing Grade 1 hemoglobin decline and 21.11% experiencing a decrease in platelet counts (Grade 1: 14.44%, Grade 2: 4.44%, Grade 3: 2.22%). Additionally, no significant differences were observed between groups with varying AUC values. All patients recovered after supportive care, which did not affect the course and dosage of treatment.

No severe Grade 3/4 hepatic, renal toxicities or cardiac dysfunctions were detected in this study. Univariate and binary logistic regression analyses revealed statistically significant differences in CREA and ALB between groups with different AUC, although the impairments were Grade 2 and below or above the normal range. However, differences in ALB may be attributed to docetaxel metabolism through the liver, which can lead to abnormalities in liver function and decreased clearance of docetaxel (Yamamoto et al., 2005). In addition, elevated levels of albumin may increase drug binding rate, reduce free drug concentration, decrease drug clearance rate, and may lead to an increase in AUC. While renal toxicity from docetaxel is rare, carboplatin may have an effect on it. We compared the incidence of CREA elevation and found that it was significantly higher (19.57%) for carboplatin-containing chemotherapy regimens than other regimens (9.70%), but this difference disappeared when patients with low CREA levels were included (28.26% vs. 28.36%). Therefore, further investigation is needed to determine whether chemotherapy-induced effects on CREA are derived from docetaxel.

With the advancement of medical humanistic care, the attention towards improving the quality of life for patients undergoing chemotherapy has increased. This study utilized a comprehensive patient adverse reaction questionnaire for collecting data on gastrointestinal reactions, canker sores, finger numbness, skin rashes, and CIA, which have frequently been disregarded in previous studies. Binary logistic regression analysis revealed that the incidence of skin rashes and CIA was significantly higher in the AUC >2.5 mg h/L group compared to the AUC ≤2.5 mg h/L group. Univariate regression analysis also showed a significant difference in vomiting. However, this difference did not remain significant in binary logistic regression analysis. These findings suggest that docetaxel TDM can help determine if these adverse reactions are due to excessive concentration and facilitate necessary adjustments to ensure chemotherapy efficacy while enhancing safety and improving patients’ quality of life.

Based on previous studies regarding chemotherapy for solid tumors in the Chinese population, this study selected an AUC range of 1.5–2.5 mg h/L for docetaxel (Sun et al., 2020). For instance, based on the administration of 75 mg/m^2^ in Chinese cancer patients, the derived safety AUC after modeling was found to be less than 2.6 mg h/L (Wei et al., 2022). Additionally, a Chinese study on head and neck cancer chemotherapy reported a safety AUC range of 2.5–3.7 mg h/L (median 2.58 mg /L) (Ma et al., 2020). In contrast, studies conducted in Spain and the Netherlands positioned the upper limits of reference values at 3.68 mg h/L (Engels et al., 2011) or 4.5 mg h/L (Aldaz et al., 2023) respectively. Although no significant racial differences were observed in docetaxel pharmacokinetics, there were higher incidences of Grade 3/4 hematological limiting toxicities among East Asian populations receiving doses between 75–100 mg/m^2^ (Kenmotsu and Tanigawara, 2015). Consequently, docetaxel dose higher than 75 mg/m^2^ was not utilized in this study. The mean AUC for patients enrolled in this study was 2.69 mg h/L, with a median value of 2.5 mg h/L. Notably, the proportion of patients in the enrolled group with AUC values greater than 2.5 mg h/L was 36.11%, exceeding our expectations, yet most individuals tolerated chemotherapy without experiencing serious adverse reactions or requiring dose reduction or course delay. Additionally, docetaxel TDM in this study employed high-performance liquid chromatography mass spectrometry, which provides greater accuracy than previous assay methods and may yield higher measurement results. The results of this study, in conjunction with the aforementioned literature reports, are not entirely consistent with the established AUC upper limit value, and differences in regional or ethnic differences, patients’ lifestyle habits, and genetic background may also affect the AUC value. Therefore, it is essential to further explore the optimal AUC range of docetaxel in Chinese breast cancer patients.

Docetaxel TDM offers a dependable foundation for adjusting the dose based on BSA. It is a routine test that is also inexpensive and accepted by patients, and the test results can be obtained before the next chemotherapy cycle. For HR (+) patients, maintaining a relative dose intensity (RDI) of chemotherapy ≥85% and ≥75% for HR (−) patients is recommended (Qi et al., 2020). In order to optimize survival benefit, it is critical to avoid arbitrary reductions of chemotherapeutic agents. Therefore, when the AUC exceeds the upper limit, docetaxel dose adjustment must be carefully decided in the context of the occurrence of adverse reactions.

The sample size in this study is limited, necessitating further research to include a larger cohort of patients and conduct multicenter studies. Additionally, it may be beneficial to collect multiple samples from the same patient at different time points to obtain a more accurate AUC measurement. It is worth considering whether local blood sampling in the body part of adverse reactions can further improve the application value of AUC. These studies will help us establish a suitable AUC reference range for docetaxel TDM for breast cancer patients in China, so as to ensure the efficacy of chemotherapy while reducing the incidence of adverse drug reactions.

In conclusion, the docetaxel TDM provides a reliable foundation for assessing chemotherapy-related adverse reactions. High AUC values are significantly associated with adverse events such as ALB, CREA, vomiting, rashes, and CIA. However, this study has several limitations. The relatively small sample size (n = 180) and the predominance of early-stage breast cancer patients (91.67% Stage I–II) may restrict the generalizability of findings to advanced populations. Furthermore, the retrospective design introduces potential confounding from heterogeneous treatment protocols and unmeasured comorbidities, despite multivariable adjustments. The short follow-up period (median <2 years) also precludes evaluation of long-term outcomes, such as survival or delayed toxicity, particularly given the absence of recurrence events in this cohort.

To enhance reliability, future studies should prioritize expanding sample sizes, prespecifying hypothesis-driven analyses (e.g., validating ALB/CREA as toxicity biomarkers), and adopting prospective designs with extended follow-up to capture longitudinal efficacy-safety dynamics. This will help overcome some of the limitations of this study and provide more robust evidence for the use of docetaxel TDM in clinical practice.

## Data Availability

The original contributions presented in the study are included in the article/Supplementary Material, further inquiries can be directed to the corresponding author.

## Abbreviations

ALB, albumin; ANC, absolute neutrophil count; AUC, area under the plasma concentration-time curve; BMI, body mass index; BSA, body surface area; CIA, chemotherapy-induced alopecia; CIN, chemotherapy-induced neutropenia; CREA, creatinine; D, dose; CL, systemic clearance; ECG, electrocardiogram; ER, estrogen receptor; FDR, false discovery rate; FN, Febrile neutropenia; HER2, human epidermal growth factor receptor 2; IPMCH, International Peace Maternity and Child Health Hospital; NTI, narrow therapeutic index; peg-G-CSF, Pegylated human granulocyte colony-stimulating factor; PR, progesterone receptor; RDI, relative dose intensity; TC, docetaxel and cyclophosphamide; TDM, therapeutic drug monitoring; TNBC, triple negative breast cancer; TKI, Tyrosine Kinase Inhibitor; UA, uric acid.
